# Effect of trajectory of employment status on all-cause mortality in the late middle-aged and older population: results of the Korea Longitudinal Study of Aging (2006-2020)

**DOI:** 10.4178/epih.e2023056

**Published:** 2023-06-08

**Authors:** Jeong Min Yang, Jae Hyun Kim

**Affiliations:** 1 Institute for Digital Life Convergence, Dankook University, Cheonan, Korea; 2 Department of Public Health, General Graduate School of Dankook University, Cheonan, Korea; 3 Department of Health Administration, Dankook University College of Health Science, Cheonan, Korea

**Keywords:** Economic stability, Korea, Mortality, Precarious employment

## Abstract

**OBJECTIVES:**

This study conducted a longitudinal analysis of the effect of trajectory of employment status (TES) on all-cause mortality in late middle-aged and older Koreans based on the Korean Longitudinal Study of Aging (KLoSA).

**METHODS:**

After excluding missing values, data on 2,774 participants were analyzed using the chi-square test and the group-based trajectory model (GBTM) for data from the first to fifth KLoSA and the chi-square test, log-rank test, and Cox proportional hazard regression for data from the fifth to eighth KLoSA.

**RESULTS:**

The GBTM analysis identified 5 TES groups: sustained white collar (WC; 18.1%), sustained standard blue collar (BC; 10.8%), sustained self-employed BC (41.1%), WC to job loss (9.9%), and BC to job loss (20.1%). Compared to the sustained WC group, the WC to job loss group had higher mortality at 3 years (hazard ratio [HR], 4.04, p=0.044), 5 years (HR, 3.21, p=0.005), and 8 years (HR, 3.18, p<0.001). The BC to job loss group had higher mortality at 5 years (HR, 2.57, p=0.016) and 8 years (HR, 2.20, p=0.012). Those aged 65 years and older and males in the WC to job loss and BC to job loss groups had an increased risk of death at 5 years and 8 years.

**CONCLUSIONS:**

There was a close association between TES and all-cause mortality. This finding highlights the need for policies and institutional measures to reduce mortality within vulnerable groups with an increased risk of death due to a change in employment status.

## GRAPHICAL ABSTRACT

[Fig f5-epih-45-e2023056]

## INTRODUCTION

Due to Korea low unemployment rate of 2% per year and high job security up to the mid-1990s, social debates and policy measures to address various health problems caused by unemployment and job changes have yet to be undertaken in great depth [1]. However, in addition to the 7% rise in Korea unemployment rate prompted by the 1997 Asian financial crisis [2], Korean companies have accelerated employment instability by restructuring to reduce labor costs, create a flexible labor market, and increase the number of non-regular and contract workers [3]. Consequently, Korea’s employment stability ranking in 2020 was the lowest among the 25 Organization for Economic Cooperation and Development countries [4], with an employee turnover rate of 5.3% in 2021, which was 5.3%, considerably higher than Japan’s 1.6% rate [5,6]. Most instances of turnover were classified as involuntary retirements, and many Koreans have reported frequent employment status changes [5-7].

Precarious employment is associated with job loss, a decline in social position, reduced income, reduced social welfare, and decreased quality of life [8,9]; in the long term, these issues can lead to deteriorated mental health and a shortened lifespan [10-12]. Numerous studies have reported an association between trajectory of employment status (TES) and negative health conditions and mortality. According to a previous study of 3,360 British workers on the relationship between TES and mental health, the subjective health level of the changed TES group was 2.18 times lower than the continuous employment status group, and the incidence of mental illness was 1.24 times higher in the changed TES group [13]. Additionally, according to a previous study of 3,764 Danish workers over a 15-year period [14], those in the changed TES group more often had anxiety disorders, a lower mental health index, and a lower energy vitality index than those in the continuous employment group. In these studies, the major causes of mental health deterioration were economic burdens and fear of involuntary retirement prompted by employment instability [13,14].

TES also influences physical health. According to a previous study that analyzed 41,728 middle-aged Japanese participants, the changed TES group had a 1.82-fold higher risk of stroke and a 3.05-fold higher mortality rate from stroke than the continuous employment group [15]. Furthermore, a previous study that included 13,451 middle-aged Americans found that the precarious employment group, which included those who had experienced 4 or more employment changes, had a 1.63 times higher incidence of myocardial infarction than the continuous employment group [16].

Although many previous studies have examined the association between TES and health status, only a few have investigated the risk of mortality related to TES. In France, the mortality rate of male precarious workers was 2.21 times higher than that of those who were continuously employed [17]. Additionally, an analysis of the correlations between mortality rates among 19,305 Korean adults revealed that the mortality rate due to diseases and factors other than diseases was 5.05 times and 4.31 times higher, respectively, in the changed TES group than in the continuous employment group [18].

However, most previous studies that demonstrated a significant association between TES and the risk of death were conducted in countries with higher employment stability than Korea. Additionally, previous findings have been limited since various occupational groups were not represented, resulting in a lack of data on the differences in TES between regular and non-regular workers or those in employed and unemployed groups. Moreover, in Korea, few studies have investigated the relationship between TES and mortality. Therefore, this study aimed to examine the effect of TES on all-cause mortality in the late middle-aged and older population using data from the Korean Longitudinal Study of Aging (KLoSA) from 2006 to 2020.

## MATERIALS AND METHODS

### Study design and source of data

The data used for the analyses were derived from the KLoSA from 2006 to 2020. All variables surveyed in the KLoSA were measured on a repeated basis from the first to fourth waves to collect data on observed cases at multiple points in time. This biennial survey involved multistage stratified sampling based on geographical areas and housing types across Korea. Participants were selected randomly using multistage stratified probability sampling to create a nationally representative sample of community-dwelling Koreans aged 45 years and older. Participant selection was performed by the Korea Labor Institute, including individuals from both urban and rural areas. When a subject withdrew his or her participation, another participant was selected from a new, similar sample from the same district [19].

**Figure 1.**
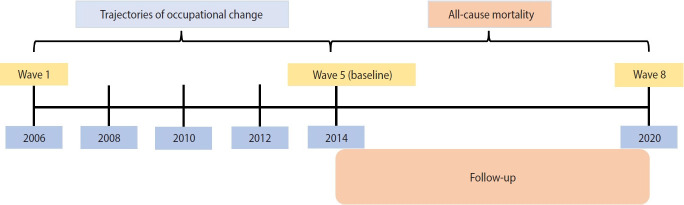
Study timeline.

Given the required measurement time of trajectory analyses, the need for a sufficient sample size, and the preference for a long follow-up time, we set the fifth wave as the baseline. Data from waves 1 to 5 (2006-2014, 8 years) were used to explore TES, and data from waves 5 to 8 (2013-2020, 8 years) were used to investigate the effect of TES on all-cause mortality. Figure 1 depicts the timeline and procedures of this study.

### Study sample

**Figure 2.**
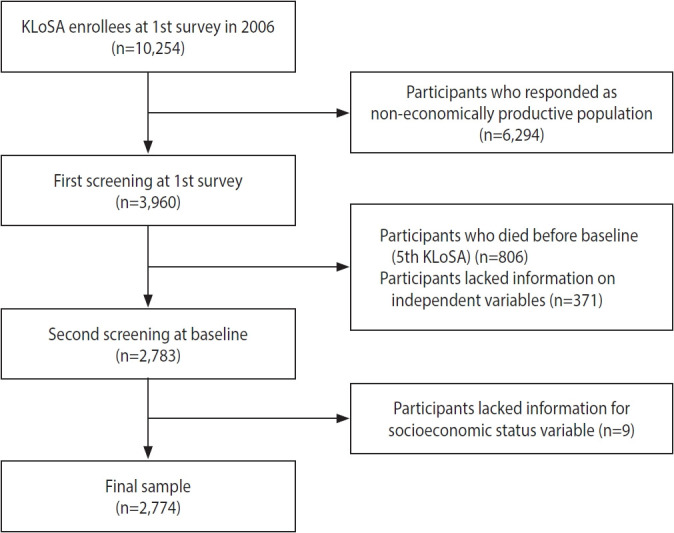
Flow chart of sample selection. KLoSA, Korean Longitudinal Study of Aging.

To investigate the effect of TES on all-cause mortality, of the 10,254 individuals registered in the KLoSA, we excluded 6,294 individuals who responded that they were non-economically productive at the time of the first KLoSA since this study aimed to identify the trajectory of labor activity among those who were workers at baseline. In addition, 806 individuals who died before baseline (fifth KLoSA), 371 individuals for whom data on independent variables were missing, and 9 individuals for whom data on control variables were missing were also excluded. Lastly, we excluded new participants in the fifth to eighth KLoSA in order to observe consistent TES data over time. We ultimately included 2,774 participants from the baseline survey in 2014 (Figure 2).

### Independent variables

#### Trajectory of employment status

Before determining the TES of the participants, job classifications of the International Labor Organization were adopted [20]. White collar (WC) workers were defined as “managers, professionals, clerical, and service workers,” while blue collar (BC) workers were defined as “sales workers, agricultural, fishing, forestry workers, craft and related trades workers, plant and machine operators, and assemblers, and elementary occupations.” We classified the participants’ occupations into 7 groups: standard WC, self-employed WC, non-standard WC, standard BC, self-employed BC, non-standard BC, and unemployed.

**Figure 3.**
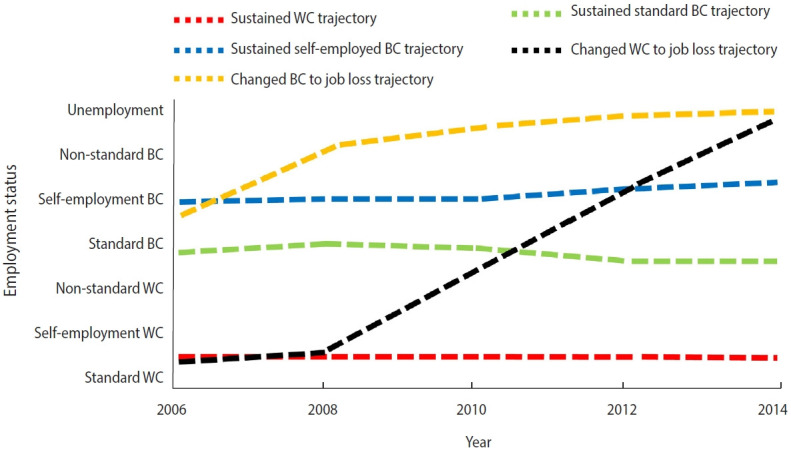
Trajectories of employment status for each group from 2006 to 2014. WC, white collar; BC, blue collar.

TES was assessed using the group-based trajectory model (GBTM) based on occupation type, resulting in 5 possible TES classifications: sustained WC, sustained standard BC, sustained self-employed BC, WC to job loss, and BC to job loss (Figure 3).

### Dependent variables

#### All-cause mortality

All-cause mortality over a maximum follow-up period of 8 years was determined if the date of death was available from the KLoSA dataset. Death information was derived from the tracker file containing exit interviews and confirmed via a certificate from the deceased patients’ spouse or partner.

### Control variables

#### Socioeconomic and demographic factors

Age was divided into 4 categories: 45-54 years, 55-64 years, 65-74 years, and 75 years or older. Sex was divided into 2 categories: male and female. Marital status was divided into 2 categories: married and single (which included separated and divorced). Education level was categorized into 4 groups: elementary school or lower, middle school, high school, and college or higher. Area of residence was categorized as urban or rural. Income level was divided into 4 categories: low, middle-low, middle-high, and high.

#### Health status and behavioral factors

The number of chronic diseases (hypertension, diabetes, cancer, chronic obstructive pulmonary disease, liver disease, cardiovascular disease, cerebrovascular disease, and arthritis) was divided into 4 groups: 0, 1, 2, and 3 or more. Activities of daily living (ADL) and instrumental activities of daily living (IADL) were divided into 3 groups: normal, mild, and impaired. Smoking status was categorized into 2 groups: smokers and non-smokers. Drinking status was also divided into 2 groups: drinkers and non-drinkers.

### Statistical analysis

The chi-square test, the log-rank test to determine the Kaplan–Meier curve, a GBTM, and Cox proportional hazards regression were used to analyze the effect of TES on all-cause mortality in late middle-aged and older Koreans.

Furthermore, this study used a GBTM to identify distinctive trajectories for the entire sample from 2006 to 2014 (first-fifth KLoSA). Trajectory modeling provides a method by which we could develop a probable representation of unobserved group classification and group differences based on observed information and user-specified constraints.

Once the TESs were derived using trajectory modeling to identify homogeneous subpopulations within the larger heterogeneous population based on the Bayesian information criterion value [21] (Supplementary Material 1), the average posterior probability of each trajectory group (> 0.85) [22] (Supplementary Material 2), and the inclusion of at least 2% of the participants within each trajectory group [23] to select distinguishable trajectories and for describing longitudinal change within each unobserved sub-population, the trajectory classes were then coded into a series of CNORM models in SAS.

A Cox proportional hazards model was used to investigate the associations between TES classifications by tracking TES classification from the first to fifth waves of the KLoSA and all-cause mortality. All Cox proportional hazards models were fully adjusted for the covariates presented in the control variables section. Furthermore, during the process of stratified analysis, adjustments were also made for variables with significant effects, such as age.

For all analyses, the criterion for statistical significance was a 2-tailed p-value < 0.05. We conducted all analyses using SAS version 9.4 (SAS Institute Inc., Cary, NC, USA).

### Ethics statement

The KLoSA study was approved by the National Statistical Office (approval No. 33602) and was conducted after acquiring the written informed consent of the participants in the study, adhering to the principles of the Declaration of Helsinki. Since the data are publicly available for scientific use with de-identified information, ethical approval was exempted for the study.

## RESULTS

### Sample characteristics

The baseline characteristics of the study participants are shown in Table 1. The mortality rates of the 2,774 participants were 1.0% (n= 27), 2.6% (n= 73), 6.1% (n= 168), and 10.1% (n= 279) at 1 year, 3 years, 5 years, and 8 years, respectively.

A total of 18.1% (n= 502) of the participants were classified in the sustained WC group for their TES, and the corresponding mortality rate was 2.8% (n= 14) at 8 years. Approximately 10.8% (n = 299) of the participants were categorized in the sustained standard BC group, and the corresponding mortality rate was 3.3% (n= 10) at 8 years. The proportion of the participants in the sustained self-employed BC group was 41.1% (n= 1,141), and their 8-year mortality rate was 11.8% (n= 135). A total of 9.9% (n= 275) of the participants were classified in the WC to job loss group, with a corresponding 8-year mortality rate of 10.5% (n= 29), and the proportion of those in the BC to job loss group was 20.1% (n= 557), with an 8-year mortality rate of 16.3% (n= 91).

**Figure 4.**
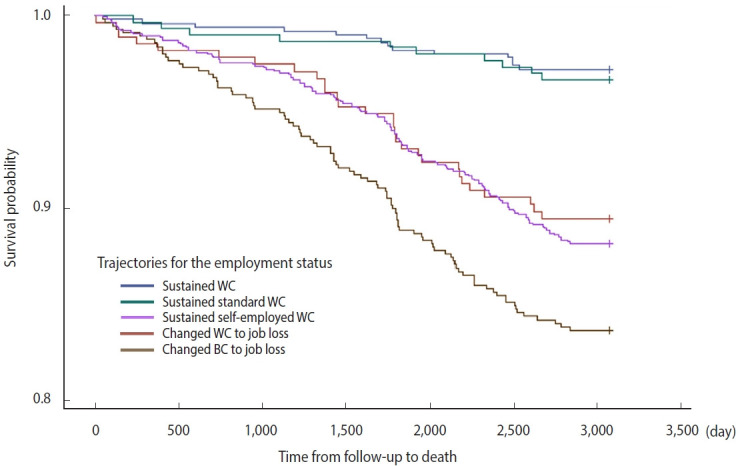
Kaplan–Meier curve for mortality. WC, white collar; BC, blue collar.

Figure 4 depicts the Kaplan–Meier curves. The results of the log-rank test showed a significant difference in the survival probability of the 5 groups related to TES (p< 0.001).

### Adjusted effect between trajectory of employment status and all-cause mortality

Table 2 shows the results of the survival analysis using a Cox proportional hazards model, which investigated the association between TES and all-cause mortality. The sustained self-employed BC group had a higher mortality rate at 8 years (hazard ratio [HR], 2.04, p= 0.018) than the sustained WC group. However, the 1-year, 3-year, and 5-year all-cause mortality rates did not show statistically significant differences.

The WC to job loss group had a higher mortality rate than the sustained WC group at 3 years (HR, 4.04, p= 0.044), 5 years (HR, 3.21, p= 0.005), and 8 years (HR, 3.18, p< 0.001). The BC to job loss group had a higher mortality rate at 5 years (HR, 2.57, p=0.016), and 8 years (HR, 2.20, p= 0.012).

### Adjusted effect between trajectory of employment status and mortality by age and sex

Table 3 shows the results of the subgroup analysis stratified by age and sex. Using the sustained WC group as a reference, we found that younger participants (≤ 64 years) in the WC to job loss group had higher mortality rates at 5 years (HR, 3.65, p= 0.008) and 8 years (HR, 2.74, p= 0.014). Older participants (≥ 65 years) in the sustained self-employed BC group (HR, 3.54, p= 0.034), WC to job loss group (HR, 5.33, p= 0.009), and BC to job loss groups (HR, 3.70, p= 0.030) had higher mortality at 8 years than the reference group.

In addition, males showed significant mortality in both the WC to job loss (5-years: HR, 3.24, p= 0.013; 8-years: HR, 3.50, p< 0.001) and BC to job loss (5-years: HR, 2.65, p= 0.027; 8-years: HR, 2.32, p= 0.017) groups at 5 years and 8 years compared to the sustained WC group. However, in the female group, no association was found between TES and all-cause mortality at 5 years and 8 years.

## DISCUSSION

We investigated the effect of TES on all-cause mortality in the late-middle-aged and older Korean population. This study found a significant relationship between TES and mortality after controlling for other factors based on a large representative sample over a 14-year follow-up period. These findings suggest that employment stability is crucial for decreasing all-cause mortality.

The major findings of our study are as follows. First, the mortality rate of the changed employment status group was higher at 3 years, 5 years, and 8 years compared to that of the sustained WC group. Second, among those aged 64 years or younger, the 5-year and 8-year mortality rates in the WC to job loss group were higher than those in the reference group. Among those aged 65 years and older, the sustained self-employed BC group, WC to job loss group, and BC to job loss group had higher 8-year mortality rates than the reference group. Third, unlike in the female subjects, changed employment status was closely related to mortality in male subjects.

The higher mortality rate of the changed TES groups compared to the continuous employment groups was consistent with the results of previous studies [24-27]. In a study that analyzed the association between changes in employment and mortality among 49,321 middle-aged and elderly people in Sweden [24], the 8-year mortality rate was 1.73-fold higher in the changed employment group than in the continuous employment group, the suicide rate was 1.98-fold higher, and the death rate due to deteriorating health was 1.47-fold higher. Furthermore, a study of 110,827 middle-aged and older adults in the United States reported that the mortality rates of the groups who lost their jobs due to employment changes and those who remained unemployed were 1.88 times and 1.33 times higher, respectively, than that of the continuous employment group [25]. According to the “coping hypothesis” [26], people who experience rapid employment changes and lose their jobs have an increase in adverse health-related behaviors that cause health deterioration owing to their precarious socioeconomic situations. Ultimately, these health behaviors can cause death by increasing the risk of mental diseases, such as anxiety disorders and dementia, as well as physical diseases, such as hypertension, cardiovascular disease, and cancer [27].

As a result of the subgroup analysis of the association between TES and mortality stratified by age in this study, a strong correlation was observed between the WC to job loss trajectory and mortality among those aged 64 years or younger, which is consistent with the findings of previous studies [28,29]. According to a previous study in Finland [28], compared to BC workers, WC workers tend to be more physically inactive, which can suppress immune function, increase workers’ susceptibility to infections, and eventually lead to death from a wide range of diseases due to high work stress. In the elderly group, regardless of occupation, TES had a significant impact on mortality. Several previous studies [30-35] have noted that the elderly are considered a vulnerable group in most countries; however, in countries where traditional family structures are common, such as Korea, continuous participation in the labor market regardless of age is often considered essential for providing family support and protecting families from income loss [30]. However, late-career unemployment and employment changes can decrease social security [31] and increase the risk of death due to the high costs of caring for dependents and household maintenance [31,32], which is not the case for those in younger age groups. In addition, changes in employment are the main cause of frailty, which reduces physical vulnerability, resistance, and functional reserves [33]. Frailty causes neuroendocrine changes, the activation of inflammatory pathways, increased insulin resistance, and decreased body energy production, thereby increasing physiological risk factors [34] as well as social risk factors, such as social isolation and loneliness, thereby increasing the risk of death [35].

Finally, employment changes had a stronger effect on mortality in the male group. These results can be attributed to the prevalence of traditional sex roles and stereotypes in Korea. Despite major recent changes in family structure in Korea, there is often a sense of expectation and duty for individuals to support the family, and males are traditionally expected to support both their children and parents [36]. Owing to these cultural characteristics, the economic activity of males is considerably more important in Korean households than that of females; therefore, sudden loss of employment is a major cause of health deterioration in males [37,38], which can lead to death in the long term [39].

However, when opportunities to participate in economic activity are offered to a vulnerable group, its overall health status and mortality rate tend to improve. A previous study in the United States examined the effect of improved mental health in an unemployed group using a re-employment intervention [40], while another study in Europe showed that, by encouraging those in age groups that face difficulties with re-employment to participate in community activities, their overall health will improve [41]. Nevertheless, health promotion programs and policies targeting these groups are inadequate in Korea [42], and the participation rate in community activities in Korea is remarkably low compared to other countries [43]. Therefore, health improvement and mortality can be enhanced through policy and institutional measures to support vulnerable TES groups.

This study has some limitations. First, this study had a subjective bias since the KLoSA data used in this analysis were combined with the opinions of the respondents. Second, although we analyzed longitudinal data, the results may reflect an inverse causal relationship. However, we made efforts to minimize the inverse correlation by adjusting for health variables (such as the number of chronic diseases and ADL/IADL) and by conducting separate analyses using the TES derivation model (2006-2014) and a model to analyze associations (2014-2020). Third, although our trajectory model was fitted based on assigned trajectories, we did not fully consider the uncertainty of class membership for each participant, which suggests that the variance estimates from this model were likely underestimated. Finally, we could not adjust for unknown confounding factors with strong associations with the investigated relationships. However, despite these limitations, our study has several strengths. First, data on the participants were assessed and analyzed over a period of 14 years. Second, the study analyzed a large representative sample, and the results can therefore be generalized to the entire middle-aged and older Korean population. Third, unlike previous studies, the analysis in this study was conducted using various types of occupations and TES classifications measured over a long period of time.

Using the survey data of the late middle-aged and older Korean population from the first to eighth KLoSA, we investigated the effect of TES on all-cause mortality.

This study’s findings highlight the need for policies and institutional measures to reduce the mortality of vulnerable groups at an increased risk of death related to TES. In particular, the mortality rate of vulnerable groups can be expected to decrease if sex and age are considered when devising new policies.

## DATA AVAILABILITY

The data analyzed in the current study are available at: https://survey.keis.or.kr/eng/klosa/databoard/List.jsp.

## Figures and Tables

**Figure f5-epih-45-e2023056:**
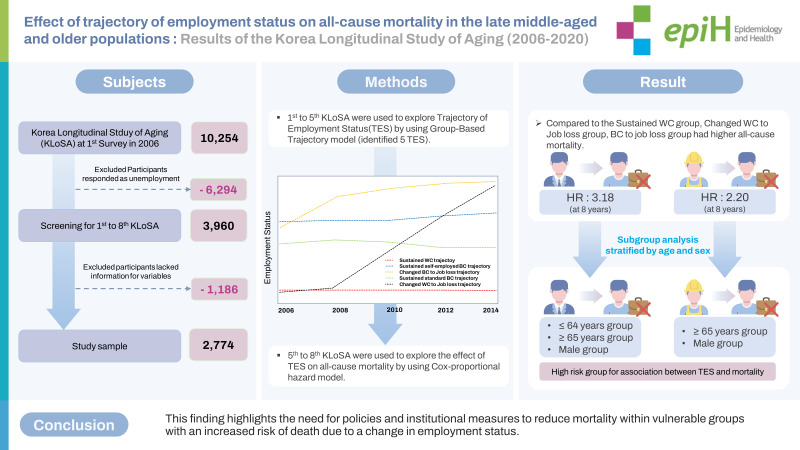


**Table 1.** General characteristics of subjects included for analysis at baseline (fifth wave of the Korean Longitudinal Study of Aging)

Characteristics		Mortality												
		Total	1-yr		p-value	3-yr		p-value	5-yr		p-value	8-yr		p-value
			Alive	Dead		Alive	Dead		Alive	Dead		Alive	Dead	
Total		2,774 (100)	2,747 (99.0)	27 (1.0)		2,701 (97.4)	73 (2.6)		2,606 (93.9)	168 (6.1)		2,495 (89.9)	279 (10.1)	
Trajectory of employment status					0.291			<0.001			<0.001			<0.001
	Sustained WC	502 (18.1)	500 (99.6)	2 (0.4)		499 (99.4)	3 (0.6)		493 (98.2)	9 (1.8)		488 (97.2)	14 (2.8)	
	Sustained standard BC	299 (10.8)	298 (99.7)	1 (0.3)		296 (99.0)	3 (1.0)		294 (98.3)	5 (1.7)		289 (96.7)	10 (3.3)	
	Sustained self-employed BC	1,141 (41.1)	1,129 (98.9)	12 (1.1)		1,108 (97.1)	33 (2.9)		1,067 (93.5)	74 (6.5)		1,006 (88.2)	135 (11.8)	
	WC to job loss	275 (9.9)	271 (98.5)	4 (1.5)		268 (97.5)	7 (2.5)		257 (93.5)	18 (6.5)		246 (89.5)	29 (10.5)	
	BC to job loss	557 (20.1)	549 (98.6)	8 (1.4)		530 (95.2)	27 (4.8)		495 (88.9)	62 (11.1)		466 (83.7)	91 (16.3)	
Age (yr)					<0.001			<0.001			<0.001			<0.001
	45-54	340 (12.3)	337 (99.1)	3 (0.9)		336 (98.8)	4 (1.2)		330 (97.1)	10 (2.9)		328 (96.5)	12 (3.5)	
	55-64	1,330 (47.9)	1,323 (99.5)	7 (0.5)		1,313 (98.7)	17 (1.3)		1,286 (96.7)	44 (3.3)		1,265 (95.1)	65 (4.9)	
	65-74	777 (28.0)	771 (99.2)	6 (0.8)		756 (97.3)	21 (2.7)		732 (94.2)	45 (5.8)		695 (89.4)	82 (10.6)	
	≥75	327 (11.8)	316 (96.6)	11 (3.4)		296 (90.5)	31 (9.5)		258 (78.9)	69 (21.1)		207 (63.3)	120 (36.7)	
Sex					0.007			<0.001			<0.001			<0.001
	Male	1,906 (68.7)	1,881 (98.7)	25 (1.3)		1,838 (96.4)	68 (3.6)		1,764 (92.5)	142 (7.5)		1,681 (88.2)	225 (11.8)	
	Female	868 (31.3)	866 (99.8)	2 (0.2)		863 (99.4)	5 (0.6)		842 (97.0)	26 (3.0)		814 (93.8)	54 (6.2)	
Marital status					0.335			0.293			0.372			0.002
	Married	2,392 (86.2)	2,367 (99.0)	25 (1.0)		2,326 (97.2)	66 (2.8)		2,251 (94.1)	141 (5.9)		2,168 (90.6)	224 (9.4)	
	Single (including separated, divorced)	382 (13.8)	380 (99.5)	2 (0.5)		375 (98.2)	7 (1.8)		355 (92.9)	27 (7.1)		327 (85.6)	55 (14.4)	
Education level					0.135			<0.001			<0.001			<0.001
	Elementary school or lower	787 (28.4)	775 (98.5)	12 (1.5)		751 (95.4)	36 (4.6)		707 (89.8)	80 (10.2)		653 (83.0)	134 (17.0)	
	Middle school	526 (19.0)	520 (98.9)	6 (1.1)		509 (96.8)	17 (3.2)		499 (94.9)	27 (5.1)		474 (90.1)	52 (9.9)	
	High school	1,021 (36.8)	1,013 (99.2)	8 (0.8)		1,006 (98.5)	15 (1.5)		979 (95.9)	42 (4.1)		954 (93.4)	67 (6.6)	
	College or higher	440 (15.9)	439 (99.8)	1 (0.2)		435 (98.9)	5 (1.1)		421 (95.7)	19 (4.3)		414 (94.1)	26 (5.9)	
Area of residence					0.465			0.016			0.039			<0.001
	Urban	1,220 (44.0)	1,210 (99.2)	10 (0.8)		1,198 (98.2)	22 (1.8)		1,159 (95.0)	61 (5.0)		1,128 (92.5)	92 (7.5)	
	Rural	1,554 (56.0)	1,537 (98.9)	17 (1.1)		1,503 (96.7)	51 (3.3)		1,447 (93.1)	107 (6.9)		1,367 (88.0)	187 (12.0)	
Income level					<0.001			<0.001			<0.001			<0.001
	Low	162 (5.8)	157 (96.9)	5 (3.1)		147 (90.7)	15 (9.3)		130 (80.2)	32 (19.8)		111 (68.5)	51 (31.5)	
	Middle-low	316 (11.4)	306 (96.8)	10 (3.2)		292 (92.4)	24 (7.6)		271 (85.8)	45 (14.2)		247 (78.2)	69 (21.8)	
	Middle-high	610 (22.0)	605 (99.2)	5 (0.8)		597 (97.9)	13 (2.1)		574 (94.1)	36 (5.9)		544 (89.2)	66 (10.8)	
	High	1,686 (60.8)	1,679 (99.6)	7 (0.4)		1,665 (98.8)	21 (1.2)		1,631 (96.7)	55 (3.3)		1,593 (94.5)	93 (5.5)	
Health insurance					0.910			0.002			0.004			<0.001
	Medical Aid	92 (3.3)	91 (98.9)	1 (1.1)		85 (92.4)	7 (7.6)		80 (87.0)	12 (13.0)		70 (76.1)	22 (23.9)	
	National Health Insurance	2,682 (96.7)	2,656 (99.0)	26 (1.0)		2,616 (97.5)	66 (2.5)		2,526 (94.2)	156 (5.8)		2,425 (90.4)	257 (9.6)	
No. of chronic diseases^1^					0.684			0.023			<0.001			<0.001
	0	1,221 (44.0)	1,211 (99.2)	10 (0.8)		1,201 (98.4)	20 (1.6)		1,175 (96.2)	46 (3.8)		1,146 (93.9)	75 (6.1)	
	1	833 (30.0)	824 (98.9)	9 (1.1)		806 (96.8)	27 (3.2)		777 (93.3)	56 (6.7)		745 (89.4)	88 (10.6)	
	2	470 (16.9)	466 (99.1)	4 (0.9)		455 (96.8)	15 (3.2)		430 (91.5)	40 (8.5)		403 (85.7)	67 (14.3)	
	≥3	250 (9.0)	246 (98.4)	4 (1.6)		239 (95.6)	11 (4.4)		224 (89.6)	26 (10.4)		201 (80.4)	49 (19.6)	
Regular exercise (wkly)					0.044			0.001			<0.001			<0.001
	No	1,861 (67.1)	1,838 (98.8)	23 (1.2)		1,799 (96.7)	62 (3.3)		1,727 (92.8)	134 (7.2)		1,639 (88.1)	222 (11.9)	
	Yes	913 (32.9)	909 (99.6)	4 (0.4)		902 (98.8)	11 (1.2)		879 (96.3)	34 (3.7)		856 (93.8)	57 (6.2)	
ADL/IADL					<0.001			<0.001			<0.001			<0.001
	Normal	2,556 (92.1)	2,537 (99.3)	19 (0.7)		2,505 (98.0)	51 (2.0)		2,430 (95.1)	126 (4.9)		2,331 (91.2)	225 (8.8)	
	Mild	176 (6.3)	173 (98.3)	3 (1.7)		167 (94.9)	9 (5.1)		151 (85.8)	25 (14.2)		141 (80.1)	35 (19.9)	
	Impaired	42 (1.5)	37 (88.1)	5 (11.9)		29 (69.0)	13 (31.0)		25 (59.5)	17 (40.5)		23 (54.8)	19 (45.2)	
Smoking status					0.003			<0.001			<0.001			<0.001
	Non-smoker	1,412 (50.9)	1,406 (99.6)	6 (0.4)		1,394 (98.7)	18 (1.3)		1,354 (95.9)	58 (4.1)		1,311 (92.8)	101 (7.2)	
	Smoker	1,362 (49.1)	1,341 (98.5)	21 (1.5)		1,307 (96.0)	55 (4.0)		1,252 (91.9)	110 (8.1)		1,184 (86.9)	178 (13.1)	
Drinking status					0.977			<0.001			<0.001			<0.001
	Non-drinker	1,328 (47.9)	1,315 (99.0)	13 (1.0)		1,279 (96.3)	49 (3.7)		1,220 (91.9)	108 (8.1)		1,159 (87.3)	169 (12.7)	
	Drinker	1,446 (52.1)	1,432 (99.0)	14 (1.0)		1,422 (98.3)	24 (1.7)		1,386 (95.9)	60 (4.1)		1,336 (92.4)	110 (7.6)	

Values are presented as number (%). WC, white collar; BC, blue collar; ADL, activities of daily living; IADL, instrumental activities of daily living.

1 Hypertension, diabetes, cancer, chronic obstructive pulmonary disease, liver disease, cardiovascular disease, cerebrovascular disease, and arthritis.

**Table 2.** Cox proportional hazard regression analysis for the association between TES and all-cause mortality

Variables		Mortality							
		1-yr	p-value	3-yr	p-value	5-yr	p-value	8-yr	p-value
TES									
	Sustained WC	1.00		1.00		1.00		1.00	
	Sustained standard BC	0.49	0.564	1.34	0.724	0.94	0.917	1.18	0.691
	Sustained self-employed BC	0.67	0.642	1.51	0.527	1.94	0.082	2.04	0.018
	WC to job loss	2.79	0.254	4.04	0.044	3.21	0.005	3.18	<0.001
	BC to job loss	0.59	0.568	1.88	0.347	2.57	0.016	2.20	0.012
Age (yr)									
	45-54	1.00		1.00		1.00		1.00	
	55-64	0.46	0.279	0.84	0.761	0.86	0.679	1.11	0.733
	65-74	0.31	0.147	0.93	0.903	0.88	0.737	1.57	0.166
	≥75	0.93	0.181	1.71	0.375	2.06	0.062	4.14	<0.001
Sex									
	Male	1.00		1.00		1.00		1.00	
	Female	0.32	0.212	0.20	0.004	0.38	<0.001	0.52	0.002
Marital Status									
	Married	1.00		1.00		1.00		1.00	
	Single (including separated, divorced)	0.50	0.395	0.80	0.608	1.21	0.438	1.41	0.060
Education Level									
	Elementary school or lower	1.00		1.00		1.00		1.00	
	Middle school	1.18	0.774	0.99	0.966	0.76	0.236	0.95	0.765
	High school	0.79	0.677	0.62	0.176	0.81	0.344	0.85	0.355
	College or higher	0.21	0.184	0.57	0.324	1.05	0.867	0.95	0.851
Area of residence									
	Urban	1.00		1.00		1.00		1.00	
	Rural	0.91	0.832	1.38	0.235	1.13	0.475	1.28	0.066
Income level									
	Low	1.00		1.00		1.00		1.00	
	Middle-low	2.21	0.212	1.41	0.352	1.12	0.646	0.96	0.826
	Middle-high	0.50	0.354	0.49	0.104	0.61	0.076	0.66	0.046
	High	0.29	0.116	0.46	0.073	0.49	0.010	0.56	0.006
Health insurance									
	Medical Aid	0.56	0.580	1.58	0.277	1.11	0.747	1.31	0.248
	National Health Insurance	1.00		1.00		1.00		1.00	
No. of chronic diseases^1^									
	0	1.00		1.00		1.00		1.00	
	1	1.00	0.998	1.37	0.297	1.35	0.142	1.26	0.157
	2	0.69	0.546	1.02	0.949	1.28	0.285	1.30	0.141
	≥3	0.91	0.887	0.80	0.598	0.98	0.953	1.22	0.314
Regular exercise (wkly)									
	No	1.92	0.262	2.09	0.035	1.64	0.016	1.64	0.002
	Yes	1.00		1.00		1.00		1.00	
ADL/IADL									
	Normal	1.00		1.00		1.00		1.00	
	Mild	1.56	0.492	1.67	0.173	2.02	0.002	1.73	0.003
	Impaired	8.42	0.001	5.72	<0.001	4.42	<0.001	2.91	<0.001
Smoking status									
	Non-smoker	0.43	0.110	0.48	0.018	0.63	0.018	0.60	<0.001
	Smoker	1.00		1.00		1.00		1.00	
Drinking status									
	Non-drinker	0.90	0.814	2.24	0.003	1.96	<0.001	1.61	<0.001
	Drinker	1.00		1.00		1.00		1.00	

Values are presented as hazard ratio. TES, trajectory of employment status; WC, white collar; BC, blue collar; ADL, activities of daily living; IADL, instrumental activities of daily living.

1 Hypertension, diabetes, cancer, chronic obstructive pulmonary disease, liver disease, cardiovascular disease, cerebrovascular disease, and arthritis.

**Table 3.** Subgroup analysis of the associations between TES and all-cause mortality stratified by age and sex

TES		Mortality			
		5-yr	p-value	8-yr	p-value
≤64 yr					
	Sustained WC	1.00 (reference)		1.00 (reference)	
	Sustained standard BC	0.69 (0.17, 2.73)	0.594	0.87 (0.31, 2.41)	0.790
	Sustained self-employed BC	1.86 (0.75, 4.64)	0.183	1.55 (0.73, 3.26)	0.253
	WC to job loss	3.65 (1.40, 9.48)	0.008	2.74 (1.22, 6.16)	0.014
	BC to job loss	1.87 (0.60, 5.83)	0.280	2.24 (0.93, 5.41)	0.074
≥65 yr					
	Sustained WC	1.00 (reference)		1.00 (reference)	
	Sustained standard BC	2.61 (0.36, 18.88)	0.343	2.76 (0.61, 12.47)	0.188
	Sustained self-employed BC	2.47 (0.58, 10.53)	0.221	3.54 (1.10, 11.43)	0.034
	WC to job loss	3.07 (0.61, 15.40)	0.172	5.33 (1.53, 18.54)	0.009
	BC to job loss	3.62 (0.85, 15.33)	0.081	3.70 (1.14, 12.03)	0.030
Male					
	Sustained WC	1.00 (reference)		1.00 (reference)	
	Sustained standard BC	1.08 (0.34, 3.48)	0.892	1.14 (0.45, 2.87)	0.782
	Sustained self-employed BC	1.78 (0.77, 4.12)	0.178	1.87 (0.96, 3.62)	0.065
	WC to job loss	3.24 (1.28, 8.22)	0.013	3.50 (1.68, 7.28)	<0.001
	BC to job loss	2.65 (1.11, 6.32)	0.027	2.32 (1.16, 4.62)	0.017
Female					
	Sustained WC	1.00 (reference)		1.00 (reference)	
	Sustained standard BC	1.08 (0.12, 11.63)	0.981	1.16 (0.19, 7.28)	0.872
	Sustained self-employed BC	2.94 (0.52, 16.61)	0.223	2.92 (0.78, 10.93)	0.111
	WC to job loss	3.47 (0.61, 19.63)	0.160	2.66 (0.66, 10.81)	0.171
	BC to job loss	1.68 (0.27, 10.43)	0.579	1.62 (0.41, 6.34)	0.490

Values are presented as hazard ratio (95% confidence interval). TES, trajectory of employment status; WC, white collar; BC, blue collar.

## SUPPLEMENTARY MATERIALS

Supplementary Material 1. Best fitting model of trajectory class

Supplementary Material 2 Average posterior probability of trajectory model

## References

[1] Cho J, Jung H (2022). Parenthood and life satisfaction in stratified labor market: evidence from Korea. Front Public Health.

[2] Hong Y, Lee K (2022). Segmented and unequal: evidence on the dual labor market and youth unemployment from the Korea youth survey. Int Rev Public Adm.

[3] Cho J, Keum J (2004). Job instability in the Korean labour market: estimating the effects of the 1997 financial crisis. Int Labour Rev.

[5] https://www.moel.go.kr/policy/policydata/view.do?bbs_seq=20221000922.

[6] Oh HS (2022). Japan’s labor market and labor-management relations in 2022. Int Labor Brief.

[7] Cho J, Lee T, Jung H (2014). Glass ceiling in a stratified labor market: evidence from Korea. J Jpn Int Econ.

[8] Moscone F, Tosetti E, Vittadini G (2016). The impact of precarious employment on mental health: the case of Italy. Soc Sci Med.

[9] Herzog AR, House JS, Morgan JN (1991). Relation of work and retirement to health and well-being in older age. Psychol Aging.

[10] Kim SS, Subramanian SV, Sorensen G, Perry MJ, Christiani DC (2012). Association between change in employment status and new-onset depressive symptoms in South Korea - a gender analysis. Scand J Work Environ Health.

[11] Costa G, Segnan N (1987). Unemployment and mortality. Br Med J (Clin Res Ed).

[12] Yur’yev A, Värnik A, Värnik P, Sisask M, Leppik L (2012). Employment status influences suicide mortality in Europe. Int J Soc Psychiatry.

[13] Ferrie JE, Shipley MJ, Stansfeld SA, Marmot MG (2002). Effects of chronic job insecurity and change in job security on self reported health, minor psychiatric morbidity, physiological measures, and health related behaviours in British civil servants: the Whitehall II study. J Epidemiol Community Health.

[14] Cottini E, Ghinetti P (2018). Employment insecurity and employees’ health in Denmark. Health Econ.

[15] Eshak ES, Honjo K, Iso H, Ikeda A, Inoue M, Sawada N (2017). Changes in the employment status and risk of stroke and stroke types. Stroke.

[16] Dupre ME, George LK, Liu G, Peterson ED (2012). The cumulative effect of unemployment on risks for acute myocardial infarction. Arch Intern Med.

[17] Khlat M, Legleye S, Falissard B, Chau N; Lorhandicap group (2014). Mortality gradient across the labour market core-periphery structure: a 13-year mortality follow-up study in north-eastern France. Int Arch Occup Environ Health.

[18] Kim JM, Son NH, Park EC, Nam CM, Kim TH, Cho WH (2015). The relationship between changes in employment status and mortality risk based on the Korea Labor and Income Panel Study (2003-2008). Asia Pac J Public Health.

[19] https://survey.keis.or.kr/eng/klosa/klosa01.jsp.

[20] https://www.ilo.org/global/topics/labour-administration-inspection/resources-library/publications/WCMS_111331/lang--en/index.htm.

[21] Jones BL, Nagin DS, Roeder K (2001). A SAS procedure based on mixture models for estimating developmental trajectories. Sociol Methods Res.

[22] Chen Y, Campbell P, Strauss VY, Foster NE, Jordan KP, Dunn KM (2018). Trajectories and predictors of the long-term course of low back pain: cohort study with 5-year follow-up. Pain.

[23] Mirza SS, Wolters FJ, Swanson SA, Koudstaal PJ, Hofman A, Tiemeier H (2016). 10-year trajectories of depressive symptoms and risk of dementia: a population-based study. Lancet Psychiatry.

[24] Lundin A, Lundberg I, Hallsten L, Ottosson J, Hemmingsson T (2010). Unemployment and mortality--a longitudinal prospective study on selection and causation in 49321 Swedish middle-aged men. J Epidemiol Community Health.

[25] Nie J, Wang J, Aune D, Huang W, Xiao D, Wang Y (2020). Association between employment status and risk of all-cause and causespecific mortality: a population-based prospective cohort study. J Epidemiol Community Health.

[26] Muntaner C, Solar O, Vanroelen C, Martínez JM, Vergara M, Santana V (2010). Unemployment, informal work, precarious employment, child labor, slavery, and health inequalities: pathways and mechanisms. Int J Health Serv.

[27] Regidor E, Ronda E, Tapia Granados JA, Pulido J, de la Fuente L, Barrio G (2019). Reversal of upward trends in mortality during the great recession by employment status at baseline in a national longitudinal study. Am J Epidemiol.

[28] von Bonsdorff MB, Seitsamo J, von Bonsdorff ME, Ilmarinen J, Nygård CH, Rantanen T (2012). Job strain among blue-collar and whitecollar employees as a determinant of total mortality: a 28-year population-based follow-up. BMJ Open.

[29] Dhungel B, Murakami T, Wada K, Gilmour S (2021). Mortality risks among blue- and white-collar workers: a time series study among Japanese men aged 25-64 years from 1980 to 2015. J Occup Health.

[30] Lee J, Kim MH (2017). The effect of employment transitions on physical health among the elderly in South Korea: a longitudinal analysis of the Korean Retirement and Income Study. Soc Sci Med.

[31] Chu WM, Ho HE, Yeh CJ, Tsan YT, Lee SH, Lee MC (2021). Late-career unemployment and risk of frailty among older adults in Taiwan: an 8-year population-based cohort study. Geriatr Gerontol Int.

[32] Breslin FC, Mustard C (2003). Factors influencing the impact of unemployment on mental health among young and older adults in a longitudinal, population-based survey. Scand J Work Environ Health.

[33] Bartley MM, Geda YE, Christianson TJ, Pankratz VS, Roberts RO, Petersen RC (2016). Frailty and mortality outcomes in cognitively normal older people: sex differences in a population-based study. J Am Geriatr Soc.

[34] Bellumkonda L, Tyrrell D, Hummel SL, Goldstein DR (2017). Pathophysiology of heart failure and frailty: a common inflammatory origin?. Aging Cell.

[35] Gale CR, Westbury L, Cooper C (2018). Social isolation and loneliness as risk factors for the progression of frailty: the English Longitudinal Study of Ageing. Age Ageing.

[36] Kim JH, Chon D (2018). Association of solidarity between adult children and older parents with cognitive decline. Geriatr Gerontol Int.

[37] Liu H, Lou VW (2017). Patterns of productive activity engagement as a longitudinal predictor of depressive symptoms among older adults in urban China. Aging Ment Health.

[38] Yoo KB, Park EC, Jang SY, Kwon JA, Kim SJ, Cho KH (2016). Association between employment status change and depression in Korean adults. BMJ Open.

[39] Chu WM, Liao WC, Li CR, Lee SH, Tang YJ, Ho HE (2016). Late-career unemployment and all-cause mortality, functional disability and depression among the older adults in Taiwan: a 12-year population-based cohort study. Arch Gerontol Geriatr.

[40] Schuring M, Robroek SJ, Otten FW, Arts CH, Burdorf A (2013). The effect of ill health and socioeconomic status on labor force exit and re-employment: a prospective study with ten years follow-up in the Netherlands. Scand J Work Environ Health.

[41] Lüschen G, Abu-Omar K, von dem Knesebeck O (2001). Sports and physical activity in the elderly: social structural context and relation to health. Soz Praventivmed.

[42] Lee SY (2012). Future directions and issues of mental health policies in Korea. Health Welf Policy Forum.

[43] Lee MJ (2012). Predictors of attitudes toward own aging among middle-aged and elderly adults-panel analysis using latent growth modeling. Korean J Soc Welf.

