# Supplementary material for: Effect of trajectory of employment status on all-cause mortality in the late middle-aged and older population: results of the Korea Longitudinal Study of Aging (2006-2020)
Source: Epidemiol Health. 2023 Jun 8;45:e2023056. doi: 10.4178/epih.e2023056 (PMC10482569; doi:10.4178/epih.e2023056)
Supplement: Supplementary Material 1. — Best fitting model of trajectory class [file epih-45-e2023056-Supplementary-1.docx]

| **Supplementary Material 1. Best fitting model of trajectory class** | | | |
| --- | --- | --- | --- |
| **Number of groups** | **Null model** | **BIC^2^** | **BIC^3^** |
| 1 | - | -26556.17 | -26571.12 |
| 2 | 1 | -25261.72 | -25265.99 |
| 3 | 2 | -24440.45 | -24446.86 |
| 4 | 3 | -23752.36 | -23760.90 |
| **5** | **4** | **-23414.72** | **-23426.81** |
| 6 | 5 | -23776.75 | -23789.57 |
| 7 | 6 | -26556.17 | -26571.12 |
| BIC^2^= Bayesian information criterion (for the total number of participants) / N=2,774 | | | |
| BIC^3^= Bayesian information criterion (for the total number of observations) / N=11,520 | | | |
