# Supplementary material for: Effect of trajectory of employment status on all-cause mortality in the late middle-aged and older population: results of the Korea Longitudinal Study of Aging (2006-2020)
Source: Epidemiol Health. 2023 Jun 8;45:e2023056. doi: 10.4178/epih.e2023056 (PMC10482569; doi:10.4178/epih.e2023056)
Supplement: Supplementary Material 2 — Average posterior probability of trajectory model [file epih-45-e2023056-Supplementary-2.docx]

| **Supplementary Material 2. Average posterior probability of trajectory model** | | | | | |
| --- | --- | --- | --- | --- | --- |
| Average Posterior probability | Groups for occupation change trajectory | | | | |
|  | Group 1 | Group 2 | Group 3 | Group 4 | Group 5 |
|  | 0.90 | 0.94 | 0.86 | 0.90 | 0.87 |
